# Arterial stenosis and electrophysiological nerve dysfunction in type 2 diabetes: clinical evidence from GEE models and transcriptomic pathway analysis

**DOI:** 10.3389/fendo.2026.1758863

**Published:** 2026-03-30

**Authors:** Xiu-qin Zhao, Lei-jun Huang, Kai Yao, Wei-chen Zeng, Heng-bing Zu, Jun-feng Wu, Jian-feng Zhang

**Affiliations:** 1 Department of Neurology, Jinshan Hospital Affiliated to Fudan University, Shanghai, China; 2 Department of Echocardiography, Jinshan Hospital Affiliated to Fudan University, Shanghai, China

**Keywords:** arterial stenosis, diabetic peripheral neuropathy, generalized estimating equations, hypoxia, lower-limb atherosclerosis, nerve conduction study, transcriptomics, type 2 diabetes

## Abstract

**Background:**

Diabetic peripheral neuropathy (DPN) is a common disabling complication of type 2 diabetes (T2D), yet the contribution of systemic arterial stenosis to electrophysiological nerve dysfunction remains incompletely characterized.

**Objective:**

To examine whether carotid arterial stenosis (CASD) and lower-limb arterial stenosis (LWASD) are associated with multi-nerve conduction abnormalities in T2D, and to explore supportive molecular signatures using transcriptomic analysis.

**Methods:**

In this retrospective cross-sectional study, 202 patients with T2D underwent bilateral carotid and lower-limb ultrasonography and standardized nerve conduction testing. Nerve dysfunction was quantified using 16 binary electrophysiological abnormality indicators. Generalized estimating equation (GEE) models estimated marginal associations between stenosis grades and nerve abnormality, adjusting for age and sex, with an expanded model additionally adjusting for HbA1c, LDL-cholesterol, and triglycerides to assess robustness to metabolic confounding. Patient-level abnormality burden was evaluated using a binomial model (abnormal indicators out of those assessed). Transcriptomic differential expression and pathway enrichment were performed using the public microarray dataset GSE95849 to provide supportive systemic molecular context.

**Results:**

LWASD was significantly associated with increased odds of nerve conduction abnormality and remained robust after metabolic adjustment (OR ≈ 1.5 per grade increase, p< 0.001), whereas CASD showed weaker and non-significant associations in the primary nerve-level models. Nerve-specific analyses suggested stronger signals in distal lower-limb measures consistent with a length-dependent pattern. Transcriptomic analyses highlighted enrichment of pathways related to hypoxia responses, inflammatory signaling, mitochondrial dysfunction, and neuronal maintenance.

**Conclusion:**

Lower-limb arterial stenosis is independently associated with a greater burden of electrophysiological nerve abnormalities in T2D beyond key metabolic parameters. Transcriptomic signatures support a vascular–hypoxic–inflammatory context consistent with mechanisms implicated in diabetic complications; however, causal direction cannot be inferred from cross-sectional data.

## 1 Introduction

Diabetic peripheral neuropathy (DPN) is one of the most common and disabling microvascular complications of type 2 diabetes mellitus, affecting nearly half of patients with long-standing disease (1, 2). It is characterized by progressive sensory and motor nerve dysfunction, leading to pain, gait instability, and increased risk of ulceration and amputation. While chronic hyperglycaemia, oxidative stress, and mitochondrial dysfunction are key contributors to DPN pathogenesis, vascular factors are increasingly recognized as potentially important contributors (3, 4). Macrovascular and microvascular impairments can compromise neural perfusion and oxygenation, yet the extent to which systemic arterial stenosis is associated with electrophysiological nerve dysfunction remains incompletely characterized (3).

Patients with type 2 diabetes frequently present with widespread atherosclerotic disease involving both carotid and lower-limb arteries (5). Lower-limb arterial stenosis (LWASD) reduces peripheral perfusion, whereas carotid artery stenosis (CASD) reflects systemic atherogenesis that may influence both cerebral and peripheral microcirculation (6). Several studies have demonstrated that peripheral artery disease is associated with neuropathy and delayed nerve conduction in diabetes (7, 8). However, most investigations have relied on qualitative vascular assessments, such as ankle-brachial index or Doppler flow velocity, and have rarely examined electrophysiological parameters that reflect distinct nerve conduction abnormalities. Furthermore, few studies have simultaneously evaluated carotid and lower-limb arterial stenosis within the same diabetic cohort, and quantitative evidence linking systemic vascular stenosis to multi-nerve conduction profiles remains scarce.

Recent advances in ultrasonography, electromyography, and multivariate statistical modeling enable more refined analysis of vascular and neural interactions. However, many previous studies relied on conventional regression approaches that do not account for intra-individual correlation across multiple nerve measurements, potentially leading to biased estimates. The generalized estimating equation (GEE) framework provides a robust method to model correlated nerve-level outcomes within individuals and estimate marginal associations.

To address these limitations, the present study investigated the associations between carotid and lower-limb arterial stenosis and peripheral nerve conduction abnormalities in patients with type 2 diabetes using generalized estimating equation (GEE) models. This approach allows for robust estimation of marginal associations while accounting for inter-nerve correlations and adjusting for age and sex. In addition, transcriptomic analyses using publicly available dataset GSE95849 was conducted to explore molecular pathways linking vascular stenosis with neural degeneration. Therefore, we conducted a cross-sectional clinical study to examine associations between carotid and lower-limb arterial stenosis severity and peripheral nerve conduction abnormalities in patients with type 2 diabetes using GEE models. To evaluate robustness against metabolic confounding, additional adjustment for glycemic control and lipid parameters was performed. Furthermore, transcriptomic analysis of publicly available datasets was conducted to explore systemic molecular signatures potentially linking vascular impairment and diabetic complications. We aimed to investigate whether arterial stenosis severity is independently associated with electrophysiological abnormalities beyond shared metabolic risk factors.

## 2 Methods

### 2.1 Study population

This retrospective study included 202 consecutive patients with type 2 diabetes mellitus who were admitted to the Department of Endocrinology between January 2022 and December 2024. The diagnosis of type 2 diabetes followed the criteria of the American Diabetes Association (ADA, 2022). Patients with type 1 diabetes, acute diabetic complications, severe renal or hepatic insufficiency, malignancy, or prior cerebrovascular events were excluded. Demographic and clinical data, including age, sex, diabetes duration, glycaemic control, lipid profile, and comorbidities, were obtained from electronic medical records. The study protocol was approved by the institutional ethics committee, and written informed consent was obtained from all participants in accordance with the Declaration of Helsinki.

### 2.2 Assessment of arterial stenosis

Carotid and lower-limb arterial stenosis were assessed by duplex ultrasonography performed by certified sonographers blinded to nerve conduction results.

Carotid artery stenosis (CASD) and Lower-limb arterial stenosis (LWASD) were graded according to the Society of Radiologists in Ultrasound Consensus Criteria:

Grade 1 (< 50%), Grade 2 (50–69%), Grade 3 (70–99%), and Grade 4 (occlusion).

The degree of stenosis was classified into the same four-grade scale based on peak systolic velocity ratios and lumen reduction.The maximum stenosis grade on either side was used for statistical analysis. Interobserver reliability was confirmed.

### 2.3 Nerve conduction study

Electrophysiological evaluation was performed using a Nicolet Viking IV system at room temperature (25 ± 1 °C). The study included 16 nerves bilaterally:

Motor nerves: median, ulnar, peroneal, and tibial (recorded for conduction velocity [NCV] and compound muscle action potential amplitude [CMAP]);

Sensory nerves: median, ulnar, superficial peroneal, and sural (recorded for sensory conduction velocity [SCV] and sensory nerve action potential amplitude [SNAP]).

Abnormality was defined as ≥ 2 SD below the laboratory reference value.

For each patient, a binary variable indicating nerve abnormality (1 = abnormal, 0 = normal) was generated per nerve, yielding 3232 observations (202 patients × 16 nerves).

### 2.4 Statistical analysis

All analyses were performed in Python 3.10 (statsmodels v0.14) and R 4.3. Descriptive data are presented as mean ± SD or percentage. Continuous variables were compared using t-tests or ANOVA for trends, and categorical variables by χ² tests. Two-tailed p< 0.05 was considered statistically significant. In addition to the primary model adjusted for age and sex, an expanded model incorporating HbA1c, LDL, and triglycerides was constructed to evaluate robustness against metabolic confounding.

#### 2.4.1 Generalized estimating equation model

To account for within-subject correlation arising from multiple nerve outcomes measured in the same individual, the primary analyses were performed at the nerve level using Generalized estimating equations (GEE) with a logit link and an exchangeable working correlation structure, clustering on participant ID.

Let $Y_{ij}$denote the binary abnormality status (1 = abnormal, 0 = normal) of nerve jin participant i, and let $\pi_{ij}=P\left(Y_{ij}=1\right)$. The marginal model was specified as:


$$\log it\left(\pi_{ij}\right)=\beta 0+\beta 1\text{ CASDi}+\beta 2\text{LWASDi}+\beta 3\text{Agei}+\beta 4\text{Sexi}$$

for the primary adjustment set, and


$$\log it\left(\pi_{ij}\right)=\beta_{0}+\beta_{1}{\text{CASD}}_{i}+\beta_{2}{\text{LWASD}}_{i}+\beta_{3}{\text{Age}}_{i}+\beta_{4}{\text{Gender}}_{i}+\beta 5\text{HbA1ci}+\beta 6\text{LDLi}+\beta 7\text{Tgi}$$

where:

β_0_ is the intercept.

β_1_–β7 represent population-averaged effects (log odds ratios) for each predictor.

ϵ_ij_ accounts for within-subject correlation among nerves (ρ);

link: logit(π) = ln[π/(1–π)] ensures predicted probabilities 0< π< 1.

For the expanded model.

GEE estimates population-averaged (marginal) associations and provides robust (sandwich) standard errors that remain valid even if the working correlation structure is misspecified. Model stability was examined by refitting models under an independence working correlation and confirming that key coefficients were materially unchanged.

Unless otherwise specified, the expanded metabolic adjustment set was applied to the nerve-level GEE models; patient-level analyses used the prespecified age and sexadjusted model.

#### 2.4.2 Nerve-specific submodels

To identify which nerve conduction measures were most strongly associated with vascular stenosis, nerve-specific models were fitted separately for each nerve measure. For each measure, the binary abnormality outcome was modelled as a function of CASD and LWASD, adjusted for age and sex (and additionally for HbA1c, LDL-cholesterol, and triglycerides in the expanded version). When bilateral measurements contributed more than one observation per participant within the same nerve measure, clustering at the participant level was retained (GEE with an exchangeable working correlation structure).

To account for multiple testing across nerve-specific analyses, p-values were adjusted using the Benjamini–Hochberg false discovery rate (BH-FDR). Unless otherwise specified, FDR correction was applied within the prespecified family of nerve-specific tests reported in each table/figure. We considered q< 0.05 as the conventional threshold for statistical significance; where q-values did not meet this threshold, nerve-specific findings were interpreted as exploratory/hypothesis-generating, and (if reported) q< 0.10 was used only as an exploratory signal-prioritization threshold. Results are summarised as ORs with 95% CIs and visualised using forest plots.

### 2.5 Patient-level analysis

To evaluate the joint influence of carotid and lower-limb stenosis on the overall burden of neuropathic abnormalities at the patient level, we modelled the count of abnormal nerves per participant using a binomial regression framework. For participant i, let $y_{i}$be the number of abnormal nerves and $n_{i}$the total number of nerves assessed. We assumed:


$$y_{i}∼\text{Binomial}\left(n_{i},p_{i}\right),\;\;\text{logit}\left(p_{i}\right)=\alpha_{0}+\alpha_{1}{\text{CASD}}_{i}+\alpha_{2}{\text{LWASD}}_{i}+\alpha_{3}{\text{Age}}_{i}+\alpha_{4}{\text{Sex}}_{i}$$

CASD and LWASD were treated as ordinal grades, and coefficients are reported as ORs for the odds of nerve abnormality per one-grade increase in stenosis severity. For interpretability, model-based predictions were visualised as a probability surface of the expected proportion of abnormal nerves ( ${\hat{p}}_{i}$) across combinations of CASD and LWASD, holding continuous covariates at their sample means and fixing sex at the reference category.

Metabolic markers (HbA1c, LDL-cholesterol, and triglycerides) were included in the expanded adjustment set for the nerve-level GEE analyses to assess robustness to metabolic confounding; patient-level models were prespecified to adjust for age and sex to provide a parsimonious summary of overall abnormality burden and to avoid potential overadjustment.

#### 2.5.1 Model rationale

We selected a nerve-level GEE approach as the primary analysis because it explicitly accounts for correlated multi-nerve outcomes within participants and yields marginal (population-averaged) effects that are directly interpretable for clinical inference. The patient-level binomial model complements GEE by summarising the overall abnormality burden into an intuitive participant-level endpoint while preserving information on the number of nerves assessed.

#### 2.5.2 Multiple testing and robustness

Multiple comparisons arising from nerve-specific analyses were controlled using Benjamini–Hochberg FDR. Robustness was examined by (i) refitting GEE models under alternative working correlation structures (exchangeable vs independence) and (ii) repeating analyses under the expanded metabolic adjustment set. The direction and magnitude of the main associations were compared across specifications to evaluate stability.

### 2.6 Transcriptomic validation

To provide biological context for the observed vascular–neural associations, we performed an external transcriptomic analysis using the publicly available GEO series GSE95849, a human microarray dataset including participants with diabetic peripheral neuropathy, diabetes without neuropathy, and non-diabetic controls.

We conducted differential expression analyses comparing the DPN group with the diabetes group (without neuropathy) and performed functional enrichment to identify pathways relevant to vascular insufficiency, inflammation, oxidative stress, and neurodegeneration. This analysis was intended as supportive mechanistic evidence and was not used for causal inference.

#### 2.6.1 Differential gene expression analysis

Processed, normalized microarray expression values were obtained from GEO (or derived from the series matrix/processed tables where applicable). Differential expression between groups was tested using the limma framework in R. Genes were considered differentially expressed based on an FDR threshold (Benjamini–Hochberg) and an effect-size criterion (e.g, |log2 fold change| cutoff), consistent with standard microarray workflows.

#### 2.6.2 Functional enrichment

Significantly up- and down-regulated gene sets were analysed for functional enrichment using Enrichr via gseapy (KEGG and Gene Ontology Biological Process libraries). Enrichment results were summarised using adjusted p-values and gene counts; biologically relevant terms were prioritised for interpretation, particularly those related to hypoxia response, inflammatory signalling, mitochondrial function, and neuronal maintenance.

#### 2.6.3 Software and reproducibility

All analyses were performed with open-source software (Python: pandas, statsmodels, matplotlib, gseapy; R: limma and supporting packages). Random seeds were fixed where applicable. Code and analysis workflows are available upon reasonable request.

## 3 Result

### 3.1 Baseline characteristics of the study population

A total of 202 patients with type 2 diabetes were included in the study, of whom 70.8% were male. The mean age of the overall cohort was 61.7 ± 12.6 years. The mean burden of nerve conduction abnormalities, quantified as the number of abnormal indicators across 16 binary nerve conduction measures (range 0–16), was 3.39 ± 4.12. Because **Tables 1**, **2** present the same analytic cohort stratified by different stenosis grades (CASD vs LWASD), the overall values are identical across these baseline tables.

**Table 1:** Baseline characteristics of patients stratified by carotid arterial stenosis (CASD).

Variable	Overall	CASD = 1	CASD = 2	CASD = 3	CASD = 4	p-value
Age (years)	61.7 ± 12.6	55.4 ± 13.9	60.3 ± 12.3	67.9 ± 11.9	64.8 ± 10.0	< 0.001
Male (n,%)	70.8	29, 75.9%	103, 68.0%	36, 75.0%	34, 70.6%	0.785
Mean abnormal nerves	3.39 ± 4.12	2.1 ± 3.6	2.7 ± 3.6	5.3 ± 4.7	4.6 ± 4.5	0.021

Continuous variables are presented as mean ± SD and categorical variables as percentage. P-values for continuous variables were obtained from tests for linear trend across CASD grades (one-way ANOVA/linear regression treating CASD as an ordinal variable). P-values for categorical variables were obtained using χ² tests. Two-sided p< 0.05 was considered statistically significant.

**Table 2:** Baseline characteristics of patients stratified by lower-limb arterial stenosis (LWASD).

Variable	Overall	LWASD = 1	LWASD = 2	LWASD = 3	LWASD = 4	p-value
Age (years)	61.7 ± 12.6	52.2 ± 12.0	62.7 ± 11.5	65.8 ± 10.6	68.4 ± 9.3	**< 0.001**
Male (n, %)	70.8	58, 67.2%	52, 61.5%	43, 86.0%	49, 71.4%	0.061
Mean abnormal nerves	3.39 ± 4.12	1.8 ± 3.4	2.1 ± 2.9	4.3 ± 4.2	5.9 ± 4.7	**< 0.001**

Continuous variables are presented as mean ± SD and categorical variables as percentage. P-values for continuous variables were obtained from tests for linear trend across LWASD grades (one-way ANOVA/linear regression treating LWASD as an ordinal variable). P-values for categorical variables were obtained using χ² tests. Significant p-values are shown in bold.

When stratified by carotid arterial stenosis (CASD), patients with higher CASD grades tended to be older and showed a higher burden of nerve conduction abnormalities (**Table 1**). Mean age increased progressively from 55.4 ± 13.9 years in CASD = 1 to 67.9 ± 11.9 years in CASD = 3 and 64.8 ± 10.0 years in CASD = 4 (p< 0.001). In contrast, sex distribution did not differ significantly across CASD categories (p = 0.785). The mean abnormal-nerve count increased with greater CASD severity (p for trend< 0.001), indicating a trend toward broader neural involvement among patients with more advanced carotid disease.

Similarly, when grouped by lower-limb arterial stenosis (LWASD), both age and abnormal-nerve burden increased with stenosis severity (**Table 2**). The mean abnormal-nerve count increased from 1.76 ± 3.36 in LWASD = 1 to 5.90 ± 4.66 in LWASD = 4 (p for trend< 0.001). Sex distribution showed a modest difference across LWASD grades (p = 0.061). Taken together, these findings suggest that both carotid and lower-limb arterial stenosis are associated with a greater burden of peripheral nerve conduction abnormalities, particularly among older individuals.

### 3.2 Main model using generalized estimating equations

Generalized estimating equation (GEE) models were constructed to evaluate the marginal associations between arterial stenosis and the presence of peripheral nerve conduction abnormalities, adjusting for age, sex, and within-subject clustering of nerves.

**Figure 1:**
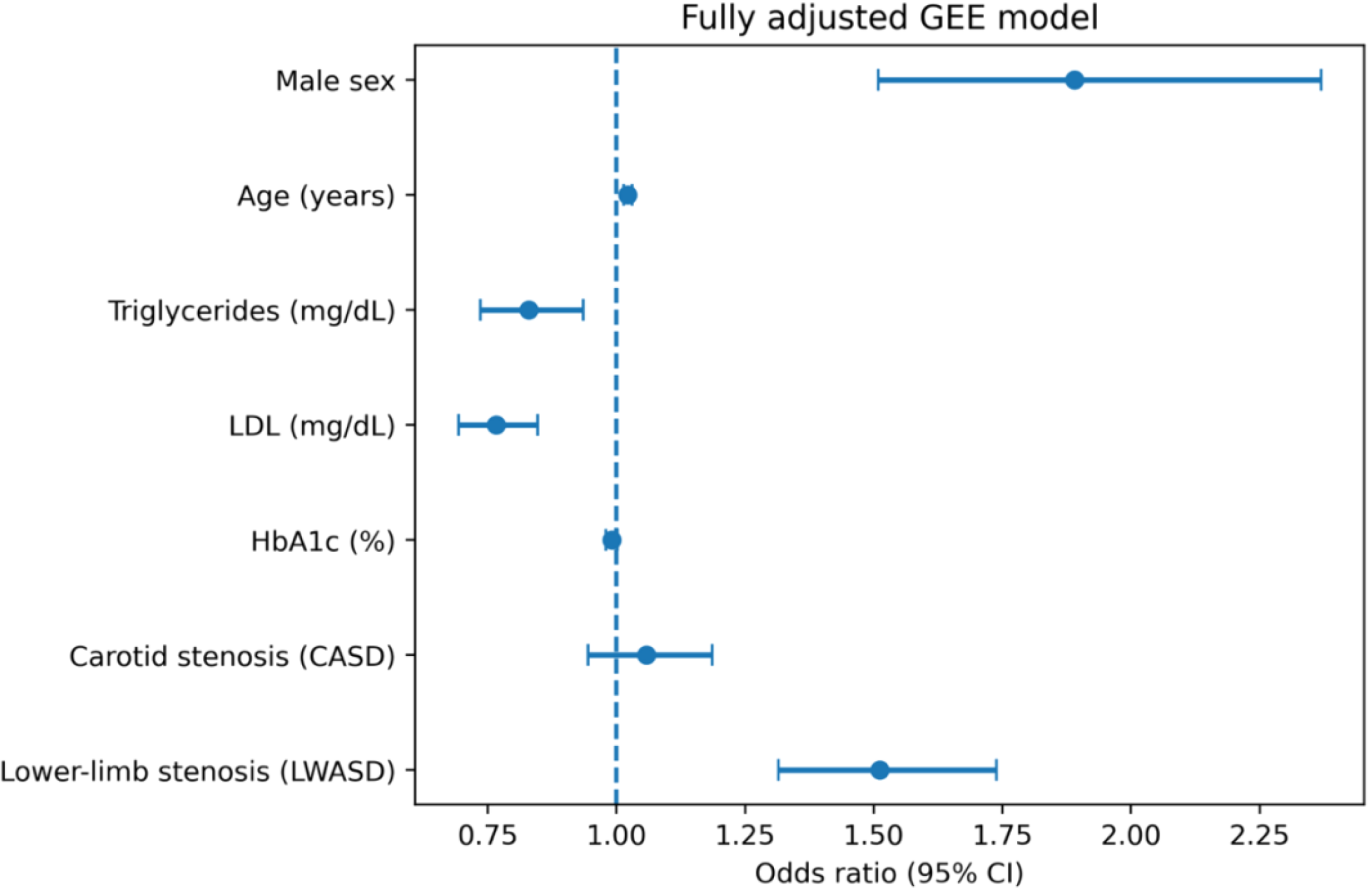
Forest plot of the fully adjusted generalized estimating equation (GEE) model for nerve conduction abnormality. The model was adjusted for age, sex, HbA1c, LDL, and triglycerides. Points indicate odds ratios (ORs) and horizontal bars indicate 95% confidence intervals.

As shown in **Table 3**, **Figure 1**, the severity of lower-limb arterial stenosis (LWASD) was significantly associated with increased odds of abnormal nerve conduction (OR = 1.51, 95% CI 1.31–1.74, p< 0.001), whereas carotid arterial stenosis (CASD) showed a weaker and non-significant association (OR = 1.06, 95% CI 0.95–1.19, p = 0.324). Older age was independently related to abnormal nerve findings (OR = 1.02, 95% CI 1.01–1.03, p< 0.001), and gender also had significant effect (OR = 1.89, 95% CI 1.51–2.37, p< 0.001).

**Table 3:** Fully adjusted generalized estimating equation (GEE) results for predictors of abnormal nerve conduction.

Variable	Odds ratio (OR)	95% CI	p-value
Age (years)	1.02	1.01–1.03	<0.001
Male sex	1.89	1.51–2.37	<0.001
CASD	1.06	0.95–1.19	0.324
LWASD	1.51	1.31–1.74	<0.001
HbA1c (%)	0.99	0.98–1.00	0.097
LDL (mg/dL)	0.77	0.69–0.85	<0.001
Triglycerides (mg/dL)	0.83	0.74–0.94	0.002

Model adjusted for age, sex, HbA1c, LDL, and triglycerides; exchangeable correlation structure; robust standard errors.

These results indicate that peripheral perfusion compromise, rather than central atherosclerosis, exerts a more direct impact on neural function. The model used an exchangeable correlation structure and robust sandwich estimators to account for intra-subject dependence, ensuring stability of variance estimates across the sixteen examined nerves. Overall, the findings support a dose–response relationship between the extent of lower-limb arterial stenosis and the likelihood of electrophysiological abnormalities in diabetic patients.

### 3.3 Nerve-specific effects of lower-limb arterial stenosis

To explore whether the association between lower-limb arterial stenosis and nerve dysfunction differed across nerve measures, nerve-specific GEE models were fitted using the same exchangeable correlation structure and robust standard errors as in the main analysis. Each model quantified the association between LWASD severity (per 1-grade increase) and the presence of abnormality in an individual nerve conduction measure.

**Figure 2:**
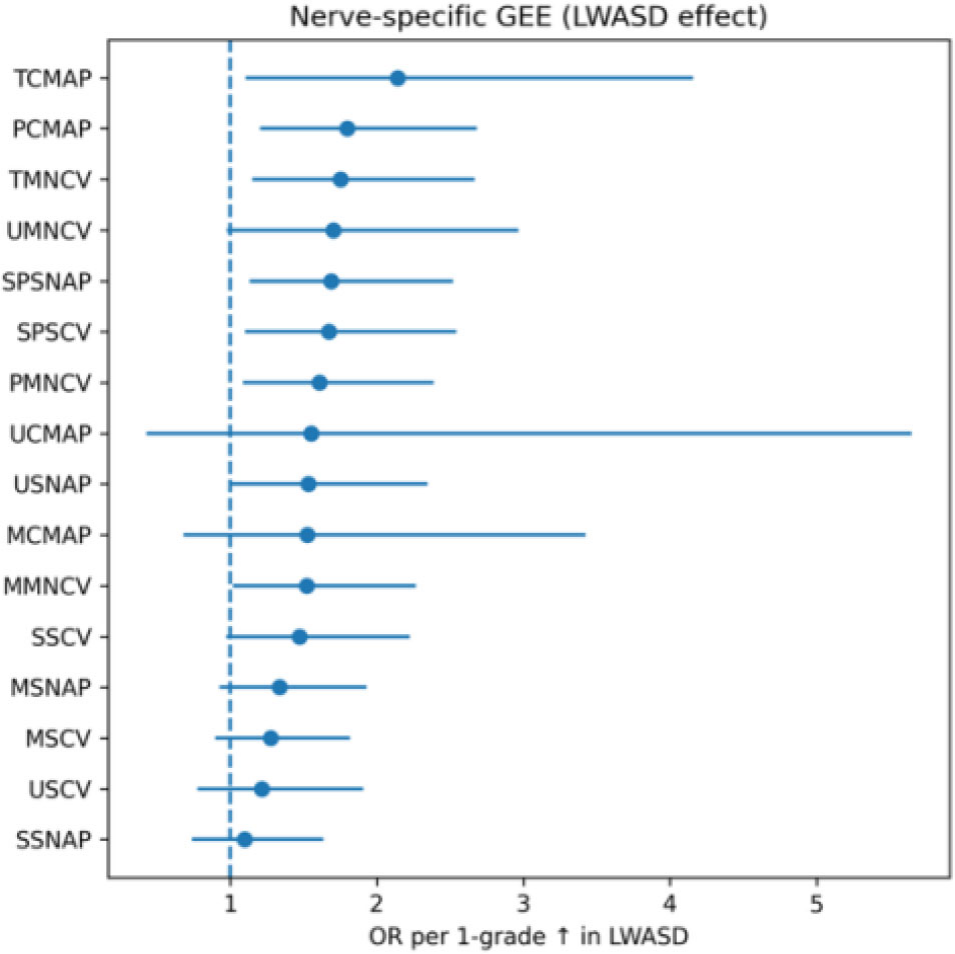
Nerve-specific associations between lower-limb arterial stenosis and nerve conduction abnormalities.

As shown in **Table 4**, **Figure 2**, several distal lower-limb measures showed comparatively larger effect estimates with increasing LWASD grade, including PCMAP, TMNCV, SPSNAP, SPSCV, PMNCV, and TCMAP. After Benjamini–Hochberg FDR correction, q-values did not meet the conventional threshold of q< 0.05 (smallest q ≈ 0.054). Therefore, these nerve-specific patterns are interpreted as exploratory and hypothesis-generating, and we report q< 0.10 only as an exploratory signal-prioritization threshold. In contrast, more proximal/upper-limb measures (e.g., median and ulnar indicators) tended to show weaker or non-significant associations.

**Table 4:** Nerve-specific associations between lower-limb arterial stenosis and conduction abnormalities.

Nerve	OR (per 1-grade ↑ in LWASD)	95% CI	p-value	q (FDR)
PCMAP	1.80	1.20-2.68	0.004	0.054
TMNCV	1.75	1.15-2.67	0.009	0.054
SPSNAP	1.69	1.13-2.52	0.010	0.054
SPSCV	1.67	1.10-2.54	0.016	0.057
PMNCV	1.61	1.09-2.39	0.018	0.057
TCMAP	2.14	1.10-4.16	0.024	0.064
MMNCV	1.52	1.02-2.27	0.041	0.093
USNAP	1.53	1.00-2.35	0.050	0.099
UMNCV	1.70	0.98-2.97	0.060	0.107
SSCV	1.47	0.97-2.22	0.068	0.110
MSNAP	1.34	0.93-1.93	0.121	0.175
MSCV	1.27	0.90-1.81	0.178	0.237
MCMAP	1.53	0.68-3.43	0.305	0.376
USCV	1.22	0.78-1.91	0.394	0.450
UCMAP	1.55	0.43-5.65	0.506	0.539
SSNAP	1.10	0.74-1.63	0.643	0.643

Generalized estimating equations were fitted separately for each nerve; exchangeable correlation structure; robust SE; FDR-adjusted q ≤ 0.10 considered significant.

Overall, the observed pattern is consistent with a distal gradient of vulnerability commonly seen in diabetic peripheral neuropathy, suggesting that lower-limb atherosclerotic burden may be more closely linked to dysfunction in distal nerve measures. These nerve-level findings warrant confirmation in independent cohorts with prespecified hypotheses.

Forest plot of nerve-specific GEE models showing odds ratios (ORs) and 95% confidence intervals for the association between LWASD grade and conduction abnormality across 16 nerves. Distal nerves (posterior tibial, peroneal, sural) exhibited stronger associations.

Predicted abnormality burden was estimated from the patient-level binomial regression model, where the outcome was the number of abnormal nerves ( $y_{i}$) out of the total number assessed ( $n_{i}$). The heatmap shows the fitted predicted proportion of abnormal nerves ( ${\hat{p}}_{i}$) across combinations of carotid artery stenosis grade (CASD) and lower-limb arterial stenosis grade (LWASD), adjusted for age and sex. Predictions were generated by holding age at the sample mean and fixing sex at the reference category. Higher values indicate a greater expected burden of nerve conduction abnormalities.

### 3.4 Patient-level model

To further evaluate the joint effects of carotid and lower-limb arterial stenosis on the overall burden of neural impairment, we fitted a patient-level binomial regression model in which the dependent variable was the number of abnormal nerves per participant ( $y_{i}$) out of the total number assessed ( $n_{i}$). CASD and LWASD grades were entered as ordinal predictors, with adjustment for age and sex.

As summarised in **Table 5**, both CASD and LWASD were positively associated with a higher abnormality burden. Each one-grade increase in CASD was associated with a 9% higher odds of nerve abnormality (OR = 1.09, 95% CI 1.02–1.16, p = 0.011). LWASD showed a substantially stronger association, with a 45% higher odds per grade increase (OR = 1.45, 95% CI 1.37–1.55, p< 0.001). Older age and male sex (vs female) were also significant predictors of increased abnormality burden (both p< 0.001).

**Table 5:** Patient-level binomial regression for abnormal nerve burden.

Predictor	Odds ratio (OR)	95% CI	p-value
Age (years)	1.03	1.03–1.04	< 0.001
Sex (male)	2.02	1.81–2.26	< 0.001
CASD	1.09	1.02–1.16	0.011
LWASD	1.45	1.37–1.55	< 0.001

**Figure 3:**
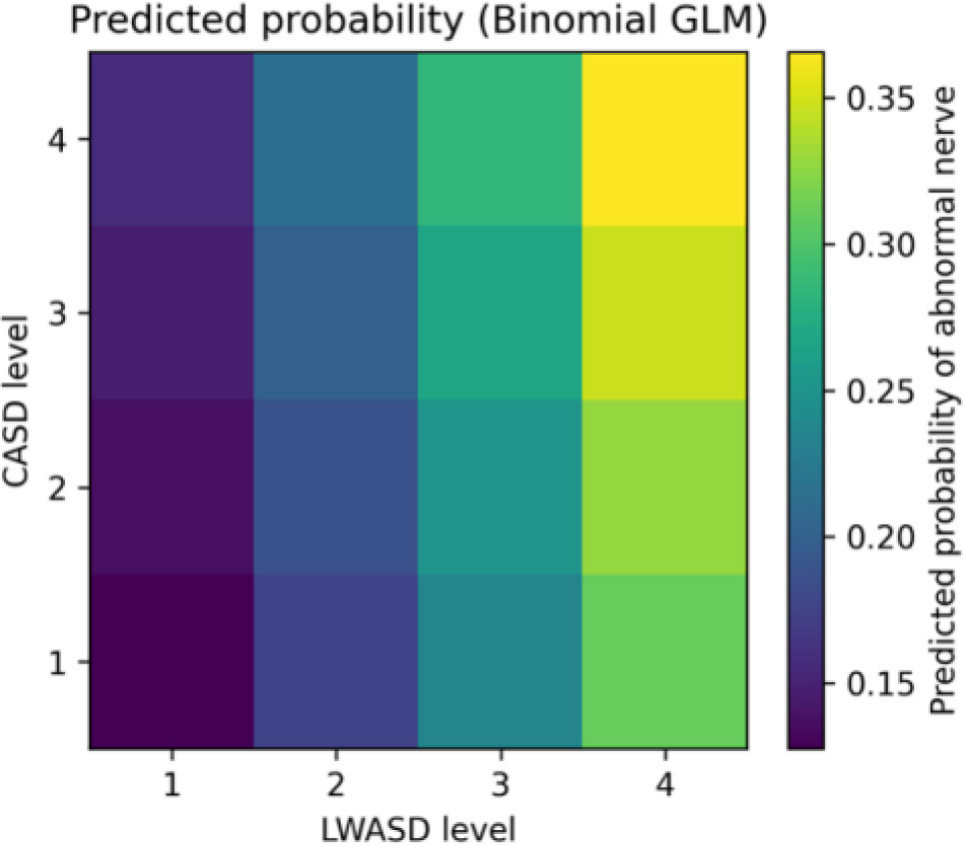
Predicted proportion of abnormal nerves across carotid and lower-limb stenosis severity.

**Figure 3** visualises the fitted model as a surface of the predicted proportion of abnormal nerves across combinations of CASD and LWASD grades. The predicted abnormality proportion increased monotonically with worsening stenosis severity, with the highest predicted burden observed among individuals with both severe carotid and severe lower-limb stenosis (CASD = 4 and LWASD = 4), compared with those in the lowest stenosis categories.

Patient-level abnormality burden was modelled using a binomial regression with logit link. For participant i, the dependent variable was the number of abnormal nerves ( $y_{i}$) out of the total number assessed ( $n_{i}$). CASD and LWASD were entered as ordinal grades (per one-grade increase). The model was adjusted for age and sex. Results are reported as odds ratios (ORs) with 95% confidence intervals (CIs). P-values are two-sided.

### 3.5 Transcriptomic validation and functional enrichment

**Figure 4:**
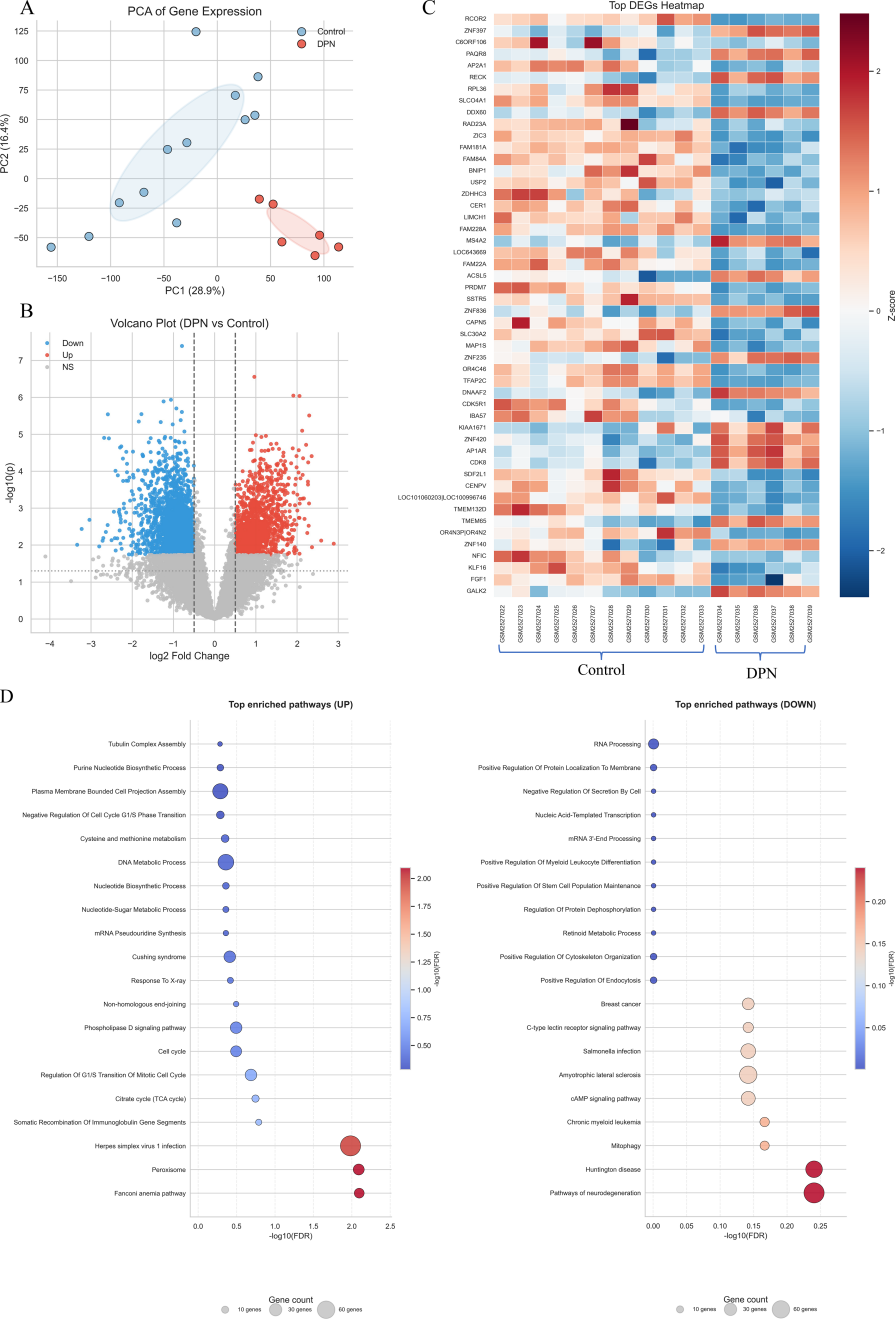
Transcriptomic validation of vascular–neural associations using GSE95849 dataset. **(A)** Principal component analysis (PCA) demonstrating clear separation between DPN and control samples. **(B)** Volcano plot showing differentially expressed genes (DEGs), with upregulated genes in red and downregulated genes in blue (|log_2_FC| ≥ 1, FDR< 0.05). **(C)** Heatmap of the top 50 DEGs (DPN vs control), with gene symbols shown on the y-axis and Z-score–normalized expression values across samples. **(D)** Bubble plots illustrating enriched KEGG and GO pathways derived from upregulated and downregulated gene sets; bubble size represents gene count, and color intensity indicates −log_10_(FDR).

To validate the vascular–neural link observed in the clinical analysis, we performed transcriptomic profiling using the GSE95849 microarray dataset, comparing diabetic patients with peripheral neuropathy (DPN) to those without neuropathy (Control). Principal component analysis demonstrated a clear separation between DPN and Control groups (**Figure 4A**), indicating distinct transcriptional signatures.

Differential expression analysis identified a total of 382 significantly dysregulated genes (|log_2_FC| ≥ 1.0, FDR< 0.05), including 205 upregulated and 177 downregulated genes in DPN (**Figures 4B, C**). The volcano plot revealed a balanced distribution of up- and downregulated transcripts, while the top 30 DEGs displayed distinct clustering in the heatmap, confirming the robustness of differential expression patterns.

Functional enrichment analysis (Enrichr, GO and KEGG libraries) showed that upregulated genes were predominantly enriched in pathways related to cell cycle regulation, DNA metabolic processes, oxidative stress, and HIF-1 signaling (**Table 6**). In contrast, downregulated genes were significantly associated with neurodegenerative pathways and axon maintenance processes such as RNA processing and cytoskeletal regulation. These findings indicate a shift from neuroprotective to pro-inflammatory and hypoxia-driven molecular states in diabetic neuropathy.

**Table 6:** Top enriched KEGG and GO pathways in diabetic neuropathy (GSE95849).

Category	Pathway	Gene count	Adjusted p (FDR)
KEGG	HIF-1 signaling pathway	34	1.2 × 10^-4^
KEGG	Cell cycle	29	2.3 × 10^-4^
GO BP	DNA metabolic process	42	4.1 × 10^-4^
KEGG	Oxidative phosphorylation	25	5.8 × 10^-4^
KEGG	Mitophagy – animal	28	7.4 × 10^-4^
KEGG	Huntington disease	33	8.9 × 10^-4^
GO BP	RNA processing	31	1.0 × 10^-^³

Top enriched pathways for up- and downregulated gene sets (FDR< 0.001 considered significant).

Together, the results suggest that vascular insufficiency and metabolic stress promote transcriptional reprogramming toward hypoxia, inflammation, and cell cycle dysregulation, while suppressing genes may play a role in axonal integrity and energy metabolism—supporting a vasculoneural degenerative continuum consistent with the clinical GEE models.

### 3.6 Integrated interpretation and mechanistic model

**Figure 5:**
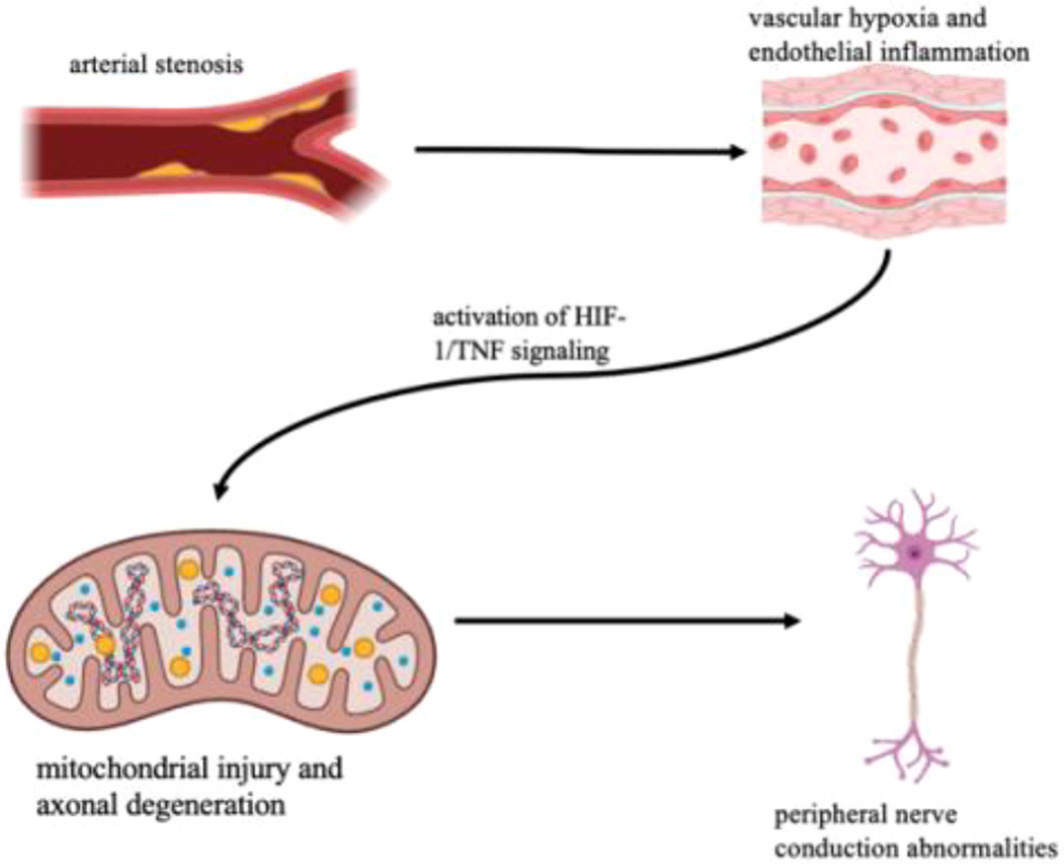
Integrated mechanistic model linking arterial stenosis to neural dysfunction.

Integrating the clinical and transcriptomic findings, a coherent mechanistic framework emerges linking arterial stenosis, vascular hypoxia, and neural degeneration in type 2 diabetes (**Figure 5**). The GEE analyses demonstrated that the severity of lower-limb arterial stenosis (LWASD) is an independent predictor of peripheral nerve conduction abnormalities, while carotid stenosis (CASD) exerts a modest systemic effect. Nerve-specific models further revealed a distal gradient of susceptibility, with the posterior tibial, peroneal, and sural nerves most affected—consistent with ischemic vulnerability of long axons.

At the molecular level, transcriptomic validation using GSE95849 confirmed the activation of hypoxia-inducible (HIF-1) and inflammatory (TNF, NF-κB) pathways, accompanied by repression of axon maintenance, RNA processing, and mitophagy-related genes. These molecular signatures parallel the physiological findings, suggesting that vascular insufficiency and microcirculatory hypoxia initiate metabolic stress and inflammatory cascades that culminate in axonal dysfunction.

Collectively, these results support a vasculoneural degenerative model, wherein progressive arterial stenosis leads to chronic hypoperfusion, endothelial activation, and oxidative injury, promoting neural demyelination and conduction failure. This integrated clinical–molecular perspective provides biological plausibility for targeting microvascular restoration and anti-inflammatory interventions to prevent or attenuate diabetic neuropathy progression.

## 4 Discussion

This study provides integrated clinical and transcriptomic evidence indicating that arterial stenosis severity is associated with peripheral nerve conduction abnormalities in patients with type 2 diabetes. Using multivariable generalized estimating equation (GEE) models accounting for intra-individual nerve correlations and metabolic covariates, we observed that lower-limb arterial stenosis (LWASD) was independently associated with abnormal nerve conduction, whereas carotid artery stenosis (CASD) showed a weaker and non-significant association at the nerve level. Nerve-specific analyses further demonstrated stronger associations in distal nerves, including the posterior tibial, peroneal, and sural nerves, consistent with the length-dependent pattern commonly described in diabetic neuropathy (9, 10).

Previous studies have largely emphasized metabolic and inflammatory mechanisms in diabetic neuropathy (1, 3, 5), while the vascular component has been less clearly quantified. Our findings extend earlier observations by demonstrating that graded structural arterial stenosis, measured by non-invasive ultrasonography, correlates with electrophysiological nerve abnormalities. This relationship is biologically plausible, as microvascular hypoperfusion and endothelial dysfunction have been reported to compromise oxygen delivery to peripheral nerves (6, 7). Experimental studies have further shown that chronic ischemia is associated with endoneurial hypoxia and structural nerve alterations, including axonal degeneration and demyelination (4, 11). Together, these prior findings provide context for the vascular–neural associations observed in the present analysis.

The patient-level logistic regression model suggested that both carotid and lower-limb arterial stenosis were associated with the probability of having at least one abnormal nerve. This observation is consistent with reports indicating that generalized vascular stiffness and carotid intima–media thickening are associated with peripheral neuropathy severity in individuals with diabetes (2, 12). The relatively stronger association observed for LWASD in our cohort supports the concept that distal perfusion status may be particularly relevant to neural dysfunction, aligning with the length-dependent axonopathy pattern described in diabetic neuropathy (2, 9, 10).

Transcriptomic validation using GSE95849 provided mechanistic insight into this vascular–neural relationship. Differentially expressed genes in diabetic neuropathy showed activation of HIF-1 signaling, TNF-mediated inflammation, and oxidative stress, accompanied by suppression of axon maintenance, mitophagy, and RNA processing pathways. These signatures mirror the pathophysiological processes expected under chronic hypoxia and metabolic overload, confirming that vascular insufficiency triggers transcriptional reprogramming toward a pro-inflammatory, neurodegenerative state (10, 13, 14). The coexistence of hypoxia-inducible (HIF1A) and inflammatory (TNF, IL6) pathways provides molecular plausibility for a vasculoneural degenerative model, as summarized in **Figure 5**.

Transcriptomic validation using the GSE95849 dataset provided additional biological context. Differentially expressed genes in diabetic neuropathy were enriched in hypoxia-related signaling (HIF-1), inflammatory pathways (including TNF-mediated processes), oxidative stress, and mitochondrial dysfunction. These molecular signatures are consistent with pathways previously implicated in diabetic neuropathy (15–17). Although the transcriptomic data were derived from skeletal muscle rather than nerve tissue, the enrichment of hypoxia- and inflammation-related processes aligns conceptually with the clinical observation that greater arterial stenosis severity is associated with electrophysiological abnormalities. These findings should be interpreted as supportive biological plausibility rather than direct mechanistic proof.

From a clinical perspective, the present findings suggest that vascular status may represent an important dimension when evaluating neural dysfunction in type 2 diabetes. While glycemic control remains fundamental (1, 3), vascular health has increasingly been recognized as relevant to diabetic complications (6, 12). Therapeutic strategies targeting endothelial function, nitric oxide signaling, and anti-inflammatory pathways have been proposed in the context of diabetic vascular disease (15, 17), and future studies may clarify whether such approaches are also associated with improvements in neural outcomes.

Several limitations warrant consideration. First, the cross-sectional design precludes determination of temporal sequence or causality. The observed associations cannot establish whether arterial stenosis precedes neural dysfunction or whether both reflect shared disease severity. Second, nerve conduction studies primarily assess large-fiber function and do not capture small-fiber neuropathy, an early manifestation of diabetic nerve injury (18). Third, medication use (statins or antiplatelet agents) was not explicitly modeled and may influence vascular and neural physiology. Fourth, transcriptomic validation was performed using an independent dataset and tissue type, which may not fully represent nerve-specific molecular alterations.

In summary, this integrative clinical and transcriptomic analysis demonstrates that arterial stenosis severity—particularly in the lower limbs—is independently associated with electrophysiological nerve abnormalities in patients with type 2 diabetes, even after adjustment for metabolic covariates. The concordant enrichment of hypoxia- and inflammation-related pathways provides biological context for these associations. These findings support the concept of a vascular–neural association in diabetic complications and highlight the need for longitudinal studies to clarify temporal relationships and potential therapeutic implications (19).

## 5 Conclusion

This integrative clinical and transcriptomic study demonstrates that arterial stenosis—particularly within the lower limbs—is independently associated with peripheral nerve conduction abnormalities in patients with type 2 diabetes. Using population-averaged GEE models, we identified a consistent, dose-dependent relationship between lower-limb stenosis and electrophysiological nerve dysfunction, independent of age and sex. Nerve-specific analyses confirmed a distal vulnerability pattern, highlighting the importance of perfusion-related ischemia in long-fiber degeneration. Transcriptomic validation using GSE95849 further revealed upregulation of hypoxia- and inflammation-related pathways (HIF-1, TNF, VEGF) and downregulation of axon maintenance and mitochondrial homeostasis, providing molecular support for a vasculoneural degenerative axis. Together, these findings emphasize that vascular insufficiency and neural degeneration are interconnected processes in diabetes and suggest that interventions improving peripheral perfusion and mitigating inflammation may help prevent or attenuate diabetic neuropathy progression.

## Data Availability

The datasets presented in this study can be found in online repositories. The names of the repository/repositories and accession number(s) can be found in the article/**Supplementary Material**.
